# The conservative treatment of congenital scoliosis with hemivertebra: Report of three cases

**DOI:** 10.3389/fped.2022.951832

**Published:** 2022-11-09

**Authors:** Matteo Caredda, Diletta Bandinelli, Francesco Falciglia, Marco Giordano, Angelo Gabriele Aulisa

**Affiliations:** ^1^Department of Aging, Neurological, Orthopedic and Head-Neck Sciences, Agostino Gemelli University Polyclinic (IRCCS), Rome, Italy; ^2^Department of Orthopedics and Traumatology, Bambino Gesù Children's Hospital (IRCCS), Rome, Italy; ^3^Department of Human, Social and Health Sciences, University of Cassino and Southern Lazio, Cassino, Italy

**Keywords:** hemivertebra, congenital scoliosis (CS), conservative treatment (CT), failure of formation, bracing

## Abstract

**Introduction:**

Scoliosis is the most common type of congenital vertebral disease. This spinal disorder may be due to a failure of formation, segmentation, or a combination thereof. Complete failure of formation causes hemivertebra which can lead to unbalanced growth and deformation. Statistically, 25% of congenital curves do not evolve, 25% progress slightly, while the remaining 50% develop quickly and require treatment. Hemivertebrae can be divided into three types: non-segmented, semi-segmented, and fully-segmented. The fully-segmented types are most likely to progress. Hemivertebra in the thoracolumbar region shows higher rates of progression compared with those in the lumbar area. The treatment may be either conservative or surgical. In general, bracing is not recommended in short and rigid curves, although it may help process secondary curves.

**Objective:**

To assess the effectiveness of bracing in congenital scoliosis due to hemivertebra.

**Cases presentation:**

Searching in our database, we found three cases of patients with congenital scoliosis due to fully-segmented hemivertebra. The first of them was 6 years old at the time of diagnosis with a fully-segmented hemivertebra in L5, determining an L1-L5 (S1) lumbar curve. The second one was 10 years old at the time of diagnosis with a fully-segmented hemivertebra in L2 and a T11-L4 (L5 sacralized) thoracolumbar curve. The last one was 3 years old at the time of diagnosis with a fully-segmented hemivertebra in L3 (in six lumbar bodies), determining a thoracolumbar curve T12-L4.

**Results:**

We utilized a Milwaukee brace for the first patient, a Boston brace for the second patient, and a Progressive Action Short Brace (PASB) for the third patient. At the beginning of the treatment, the Cobb angles measured 23°, 53°, and 25°, respectively. During treatment, the Cobb angles measured 22°, 35°, and 15°, respectively. At the end of treatment, the Cobb angles measured 18°, 45°, and 12°, respectively. At long-term follow-up, the curves measured 20°, 45°, and 12° Cobb angles, respectively.

**Conclusions:**

Comparing our cases with those found in the literature we can confirm the ability of conservative treatment to change the natural history of congenital lumbar scoliosis due to failure of formation. From our experience, in all cases of CS with hemivertebra, before considering a surgical approach, conservative treatment should be implemented as early as possible without waiting for the progressive deformation of the adjacent normal vertebrae.

## Introduction

Congenital scoliosis (CS) is a congenital spinal lateral curve caused due to developmental defects of the vertebrae that induce unbalance in the longitudinal spinal growth (1).

It is the most common congenital spinal disorder (1 in 1000 births) (2) and is considered to be associated with any fetal injury during intra-uterine spinal development. This occurs very early, from the fifth to the eighth week of gestation, and it is frequently associated with other pathological conditions, like congenital kidney disorders, congenital heart disease, and spinal cord dysraphism (3). There are three main causes of CS: failures of formation, failures of segmentation, and mixed failures. Failure of formation is the most widespread type of congenital disorder where the normal shape of the vertebra is disrupted. Complete formation failures lead to hemivertebrae with the lack of one pedicle and a part of the vertebral body, while incomplete formation failures result in a wedged vertebra. Both defects can be lateral, determining scoliosis; posterolateral, determining lordoscoliosis; dorsal, determining lordosis; anterolateral, determining kyphoscoliosis; or ventral, determining kyphosis (4, 5). Failure of segmentation results in abnormal synostosis between vertebrae. This can lead to spinal anomalies such as blocked vertebrae and unilateral bars. Mixed failures represent an undefinable mosaic of formation and segmentation defects with no defined classification (6).

In 1910, Putti was the first to distinguish three types of hemivertebra: fully segmented (a disc from either side), semi-segmented (a disc from one side, but the other side welded to the contiguous vertebra), and non-segmented (welded on both sides to the contiguous vertebrae). Putti felt that the fully segmented type was most likely to progress (7). In 1968, Winter proposed a new classification adding unsegmented bars (8), and Nasca, in 1975, analyzed 60 cases of scoliosis or kyphoscoliosis due exclusively to a hemivertebra, hemivertebrae, or a unilateral bar concomitantly with hemivertebrae and classified these into six categories. He also reported that the position of the hemivertebra or hemivertebrae and the presence of unilateral bars are the main determining factors of deformity (9).

The natural progression of congenital scoliosis is not easily predictable because it depends on a large number of factors. In a 1986 study of 104 patients, McMaster reported that four principal factors determine the degree of scoliosis: the variety of hemivertebra, their location, the number of hemivertebrae and their relations, and the age of the patient (10). He also reported that semi-segmented and non-segmented hemivertebrae generally do not demand treatment, while fully segmented hemivertebrae may need prophylactic treatment to avoid severe deformity. It is difficult to determine which congenital curves will evolve quickly. Statistically, 25% of curves do not evolve, 25% evolve slowly, and 50% show fast evolution and require treatment (11). In general, hemivertebra in the thoracolumbar region show higher rates of progression compared with those in the lumbar area (12). The treatment can be surgical or conservative, but it is tilted toward surgery as shown by the literature (2, 13). Certainly, cases with formation failures such as fully-segmented, semi-segmented, or non-segmented hemivertebrae receive a range of treatments from observation to conservative treatment with brace or early spinal surgery, while patients with specific types of segmentation defects, like unilateral unsegmented bars, will not be improved with brace treatment (14).

Most of the congenital scoliotic curves are rigid and consequently resistant to corrective actions with braces. Therefore, the main purpose of brace treatment is to prevent the evolution of secondary curves which grow up above and below the congenital main curve, causing imbalance (2).

Since the secondary curves, contrary to the main congenital curve, are normally flexible, brace treatment may have a beneficial effect on these curves, and although the primary curve can be resistant to brace treatment the stabilizing potential exists.

The purpose of the study is to assess the effectiveness of conservative treatment in congenital scoliosis due to formation failures.

## Cases presentation

Searching in our database, we found three cases of patients with congenital scoliosis due to fully-segmented hemivertebra.

**Figure 1:**
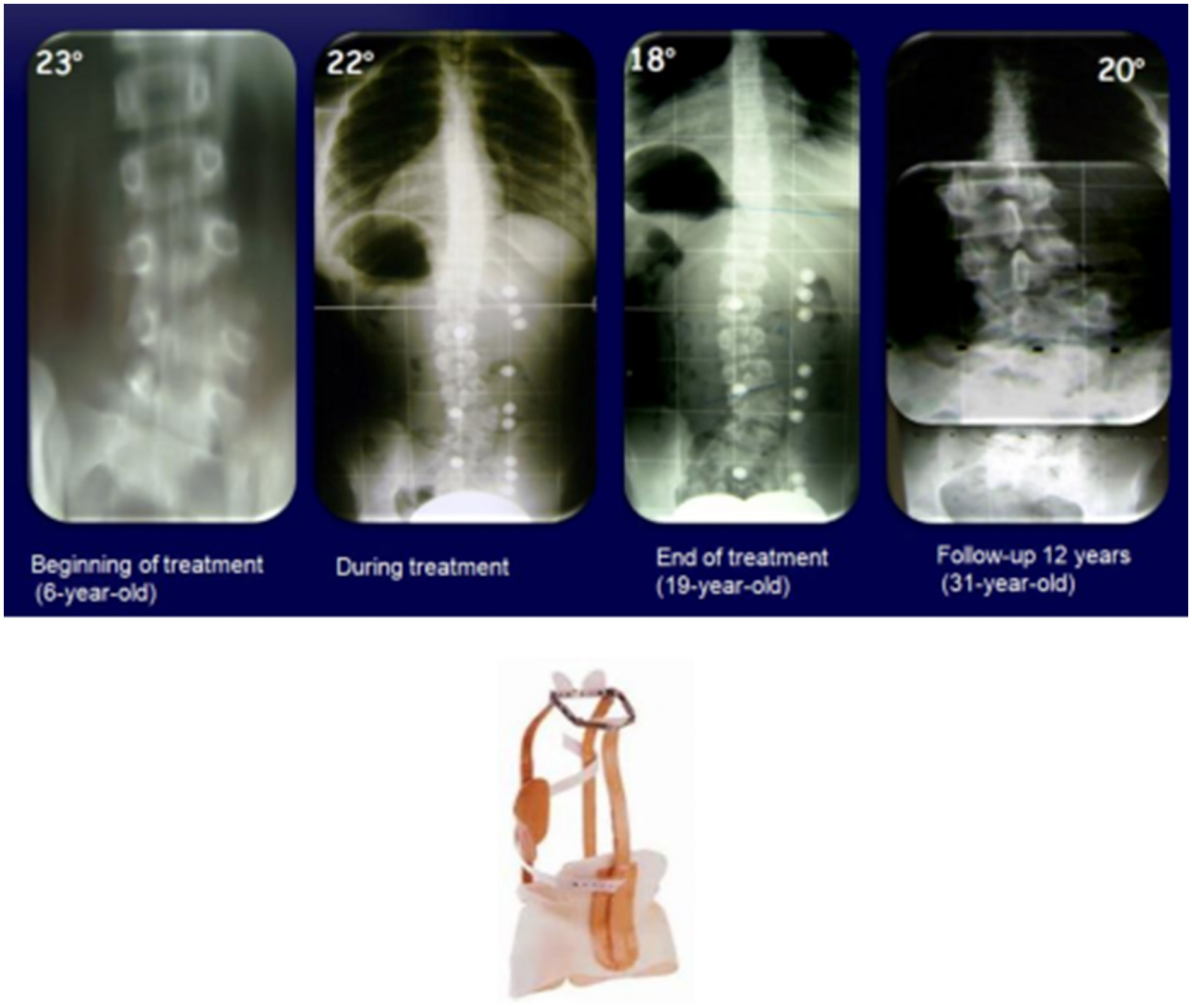
A 6-years-old girl with a hemivertebra in L5 and a curve L1-L5 (S1), with urinary malformation associated, treated with the Milwaukee brace in the first years and then the PASB.

The first case is a 6-year-old girl with a fully-segmented hemivertebra in L5 and a curve L1-L5 (S1), with associated urinary malformation. In the initial years after diagnosis, the Milwaukee brace was used and then graduated to the PASB. At the beginning of the treatment at 6 years of age, the curve was 23° Cobb, and at the weaning at 19 years of age, the curve was 18°; after 12 years of follow-up, the curve was not evolving. In particular, we noted that the vertebrae adjacent to the hemivertebra were hypertrophied (Figure 1).

**Figure 2:**
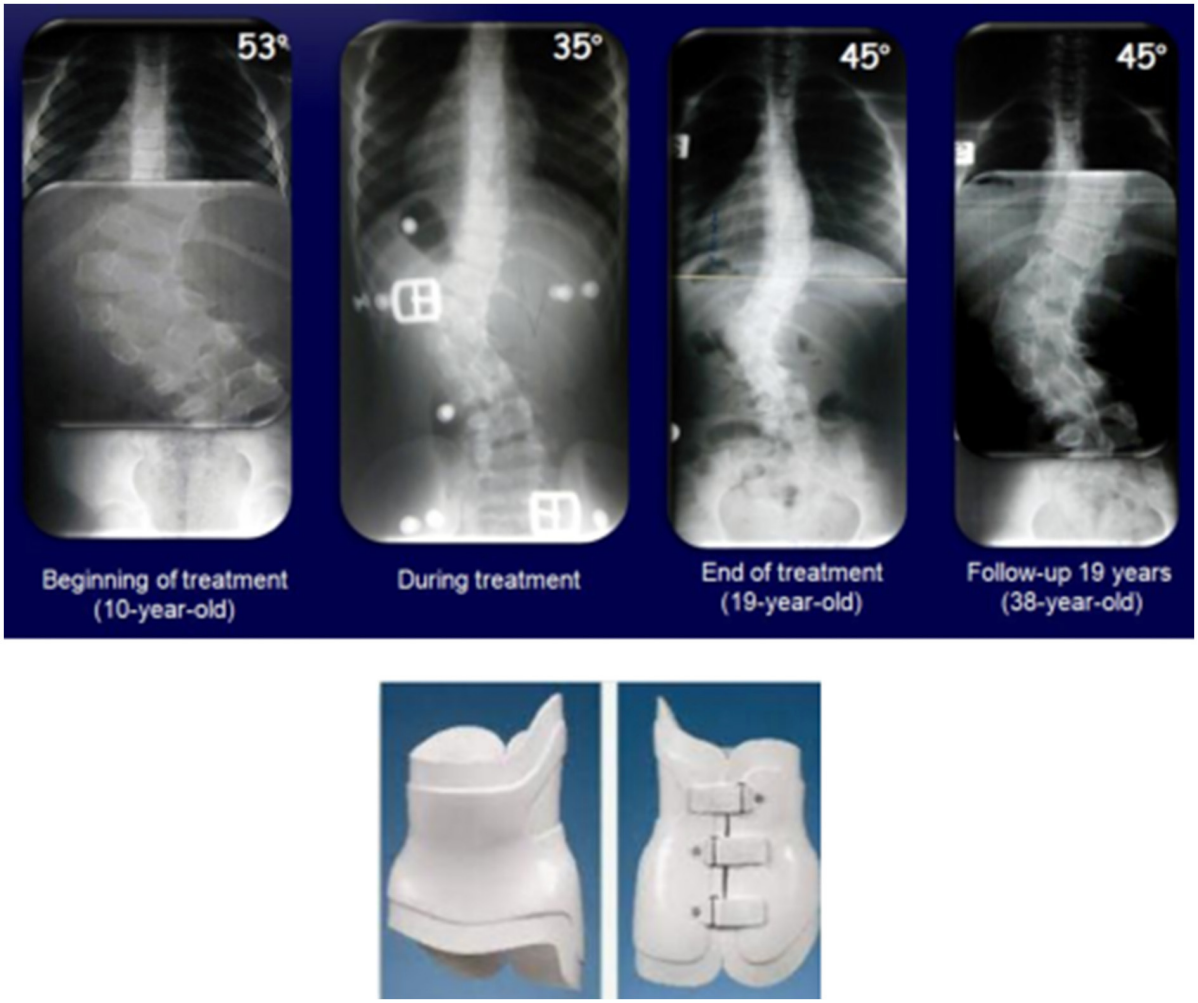
A 10-years-old female with a hemivertebra in L2 and a T11-L4 (L5 sacralized) curve treated with the Boston brace for first year and then the PASB.

The second case is a 10-year-old girl with a fully-segmented hemivertebra in L2 and a T11-L4 (L5 sacralized) curve, who refused surgical treatment. In the first year after diagnosis, a Boston brace was used, and then the PASB. At the beginning of the treatment, the curve was 53° Cobb. During treatment, the x-ray in-brace showed an improvement until 35° Cobb. At the weaning, the curve was 45° Cobb and at 19 years of follow-up, the curve was not evolving further (Figure 2).

**Figure 3:**
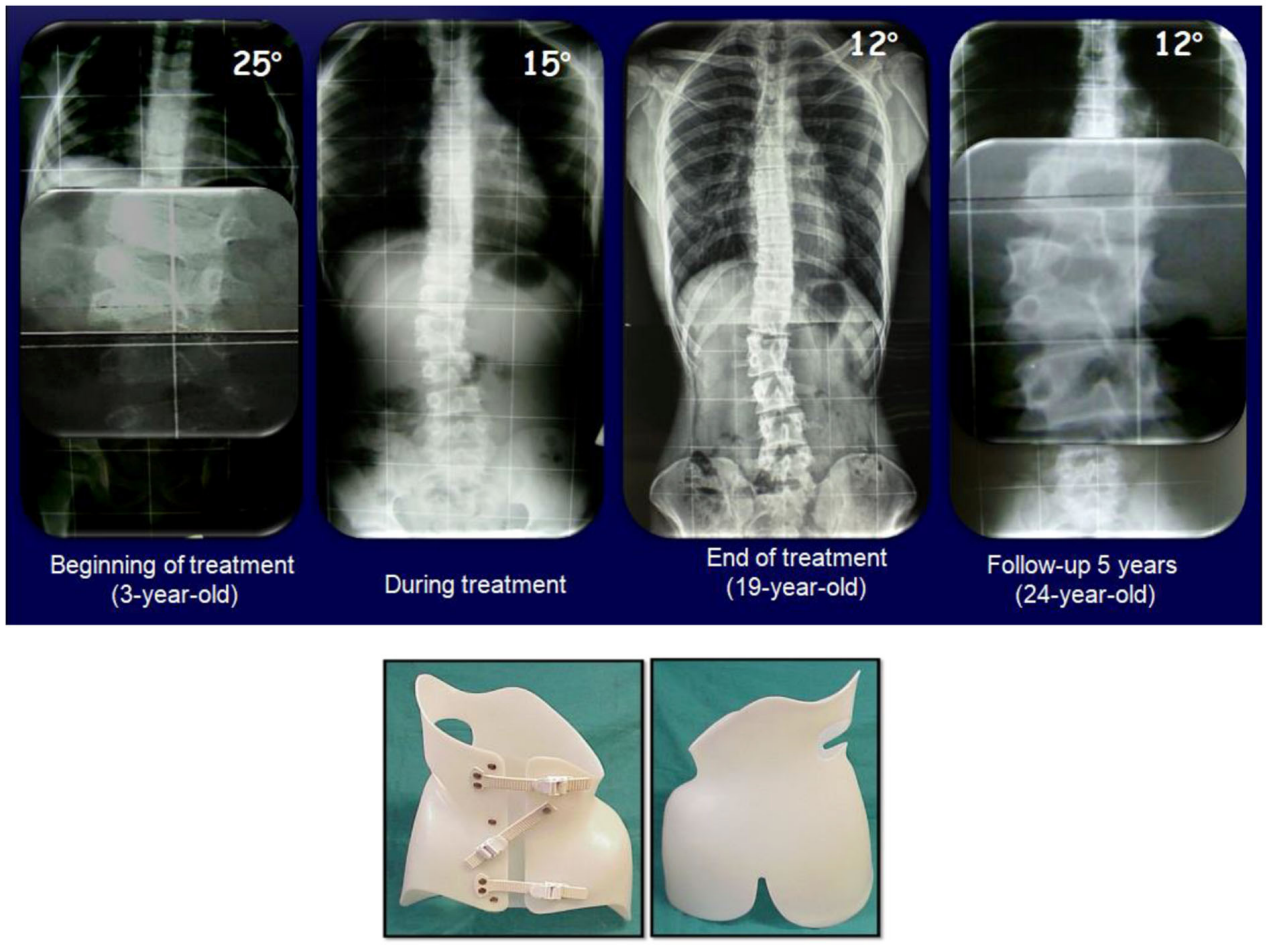
A 3-years-old male with a hemivertebra in L3 (in a 6 lumbar body) and a T12-L4 curve treated conservatively the PASB.

The third case is a 3-year-old boy with a fully-segmented hemivertebra in L3 (in a 6 lumbar body) and a T12-L4 curve. The PASB was used, and the compliance was very good. At the beginning of treatment at age three, the curve was 25° Cobb and at the weaning, the curve was 12° Cobb degrees. After 5 years of further follow-up, the curve was not evolving and the vertebrae adjacent to the hemivertebra, similar to the first case, were hypertrophied (Figure 3).

The treatment protocol for all patients consisted of full-time brace treatment, with part-time free periods based on residual growth. All three cases did not show deviations in the sagittal plane, and for this reason, we did not take into account in our study the lumbopelvic parameters and their evolution over time.

## Discussion

Treatment of spinal deformity in early childhood is difficult to manage. Literature shows that in severe cases with failures of segmentation, surgery is performed as early as possible in an attempt to control the relentless progression of the curve. Instead, in cases of failure of formation, as the ones reported, the indication of surgical or conservative treatment is not so mandatory.

These cases we have just presented show that bracing can be considered as a valid and effective treatment of CS due to hemivertebra, also considering that the fully-segmented hemivertebrae are the most likely to progress.

The age of discovery of scoliosis is an important factor (10). The three patients we have presented were treated after 2 years of age and thus were beyond the major risk of progression. However, the risk of progression in congenital curves is throughout the growth period, and starting a conservative treatment before the age of two is very difficult.

The conservative treatment is to be extended over time compared with a surgical approach. For this reason, the compliance of patients is often difficult and many prefer to undergo surgery to reduce treatment duration; however, the complication rate of surgical treatment could be greater compared with bracing treatment.

It should be noted that, to date, little data exists in the literature regarding the effectiveness of conservative treatment in CS.

In 2011, a comprehensive review was performed by Kaspiris et al., to support the assumption that early surgery is recommended in patients with congenital scoliosis. No supporting evidence for early surgical intervention was identified in this group of patients. The authors reported the effectiveness of spinal surgery in the control of deformities, but also remarked on relatively high rates of complications (up to 31%), such as implant failure, deep infection, low back pain, reduction of pulmonary function, and thoracic insufficiency syndrome (in early spinal fusion), pseudoarthrosis and neurological disorders such as neurapraxia.

Surgical indications for severe cases with rib synostosis and unilateral bar (failures of segmentation) are not in doubt. Nevertheless, formation failures frequently do not evolve and should not require surgical treatment first. For this reason, the authors concluded that, before contemplating a surgical procedure, consulting an expert in conservative management of congenital spinal malformations should be the first step looked at (2).

A Pub Med review published by Weiss in 2016 (15), as an update of the search made in 2011 with Kaspiris, was performed from 2011 to March 2015 searching for studies in support of congenital scoliosis early surgery. The Author concluded that there is no evidence (as regards randomized controlled or prospective controlled outcome studies) supporting the assumption that early surgical intervention in patients with congenital scoliosis is better than no treatment or bracing.

Chêneau et al. reported a case series of seven patients with congenital vertebral failure of formation treated conservatively with the Chêneau brace (16). They recorded an important correction of wedge angle, Cobb angle, and Chêneau index after 1 year of conservative treatment with a brace. At 2 years of follow-up, the radiographic findings had not changed significantly, but improved somewhat, concluding that bracing allows at least control and, in some cases, correction of congenital deformities of the spine.

In a case series published in 2008, Weiss reported three patients treated conservatively (two with failure of segmentation and one with failure of formation) with braces and Scoliosis In-Patient Rehabilitation (SIR) (14). In the two cases of segmentation defect, the brace treatment was at least able to avoid severe respiratory decompensation. The patient with formation failure showed balanced growth with no aesthetical or functional complaints to date, even though the curve evolved because of final poor compliance. Weiss concluded that brace treatment can be at least in part helpful in failures of segmentation, while it should be recommended first in failures of formation.

A review of the literature from 2005 to 2016 focused on surgical and conservative management of congenital scoliosis was published by Pahys et al. (17). They selected several articles in which brace treatment was not used only as an alternative to surgery, but also as a delay-tactic to surgery. They reported that considering the potential complications of impaired pulmonary function and crankshaft phenomenon related to early long spinal fusions (18) together with the complications and concerns of growth-friendly surgery (19), serial derotational bracing was described as a valid “time-buying strategy” for the treatment of congenital scoliosis (20, 21).

Another more recent study with long-term follow-up published by Weiss et al. (22) reported an 18-month-old boy with relatively balanced formation failures (hemivertebra in T7 right and another one in L1 left) and the main curve in the thoracolumbar area measuring 52° of Cobb angle. Conservative treatment with a Chêneau spinal brace began promptly and the wearing time of the brace was 18 h/day, in the beginning, and between 7–11 years it was reduced to 12 h/day because of low growth dynamics and at the inception of puberty was increased again about 20 h/day. Until age seven, the main curve steadily decreased to an angle of 40° Cobb, at 13 years, it progressed back to 50° Cobb and at 15 years it further progressed to 58° Cobb after the loss of compliance. Sporadic clinical and radiological controls were performed during 22 years of follow-up and, at the final assessment at the age of 24, the main curve of 63° Cobb was calculated.

The patient's clinical appearance was satisfying, he was normally painless and reported a good quality of life. The authors concluded that, as opposed to the conventional surgical approach, early surgical intervention for vertebral formation failures is not imperative. Patients with disorders of vertebral formation should not undergo early surgical procedures before trying an adequate treatment with a brace.

As regards Juvenile Scoliosis (JS), Canavese et al., reported encouraging results with the Elongation, Derotation, and Flexion (EDF) casting technique, in particular, if performed under general anesthesia and neuromuscular blocking drugs (23). They reported that acting simultaneously in sagittal, frontal, and axial planes, EDF casting technique can control the evolution of the deformity and, sometimes, coax the originally curved spine to straighten up, demonstrating the effectiveness of conservative treatment even in older subjects.

## Conclusions

Comparing our cases with those found in the literature we can confirm the ability of conservative treatment to change the natural history of congenital scoliosis due to failure of formation. In congenital scoliosis, aggressiveness can vary but the evolution of the congenital curve is certain. So, the treatment, given the natural progression, should be implemented as early as possible without waiting for the evolution of the curve, contrary to what occurs in lower idiopathic scoliosis. Conservative treatment, in fact, is to prevent the deformation of the adjacent vertebrae to hemivertebra that, if left to its potential deforming, induce a progressive deformation of the adjacent normal vertebrae. Once enlarged, and the deformity to the adjacent vertebrae occurs, the remodeling capacity of conservative treatment results tends to be low. Although conservative treatment with these guidelines is to be extended over time, the benefits, compared to the output and complications of surgical treatment, are evident.

## Data availability statement

The raw data supporting the conclusions of this article will be made available by the authors, without undue reservation.

## Author contributions

All authors listed have made a substantial, direct, and intellectual contribution to the work and approved it for publication.

## Conflict of interest

The authors declare that the research was conducted in the absence of any commercial or financial relationships that could be construed as a potential conflict of interest.

## Publisher's note

All claims expressed in this article are solely those of the authors and do not necessarily represent those of their affiliated organizations, or those of the publisher, the editors and the reviewers. Any product that may be evaluated in this article, or claim that may be made by its manufacturer, is not guaranteed or endorsed by the publisher.
